# Supplementary material for: Can anthropomorphic design in beverage packaging enhance impulse buying intention? Amazing visual and verbal cues!
Source: PLoS One. 2025 Jun 16;20(6):e0326186. doi: 10.1371/journal.pone.0326186 (PMC12169521; doi:10.1371/journal.pone.0326186)
Supplement: S1 Appendix — (PDF) [file pone.0326186.s001.pdf]

APPENDIX (FOR REVIEW ONLY)

These images are a collection of on-site questionnaire photographs taken by the authors about this research. The images are original and are used for review purposes only and are not intended for public distribution. The authors hope that this study will provide valuable insights and guidance for research related to the effect of anthropomorphic design in beverage packaging on consumer impulse purchase intentions.

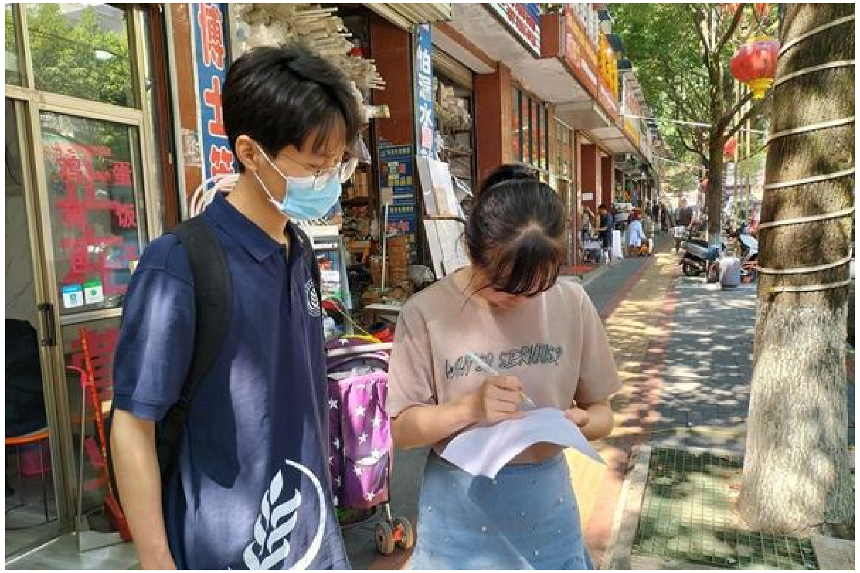

Fig 1. A team member waiting for a female participant to fill out a questionnaire.

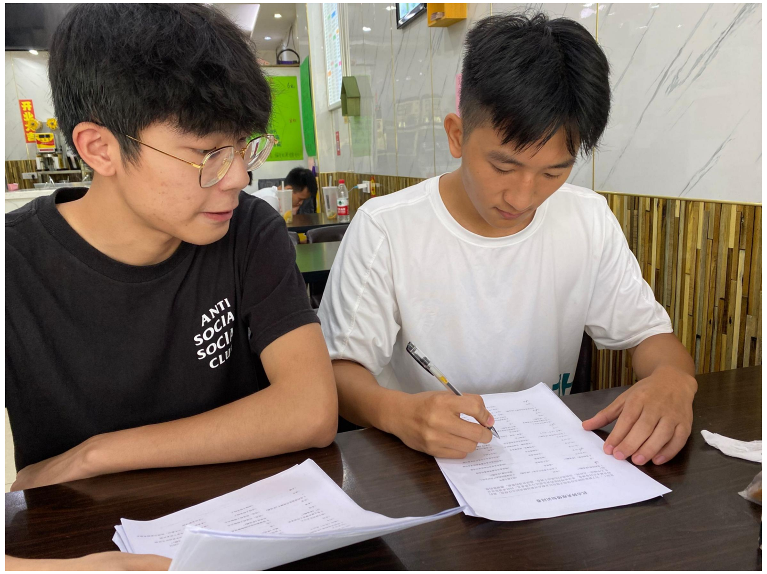

Fig 2. A team member is explaining the questionnaire to a male participant.

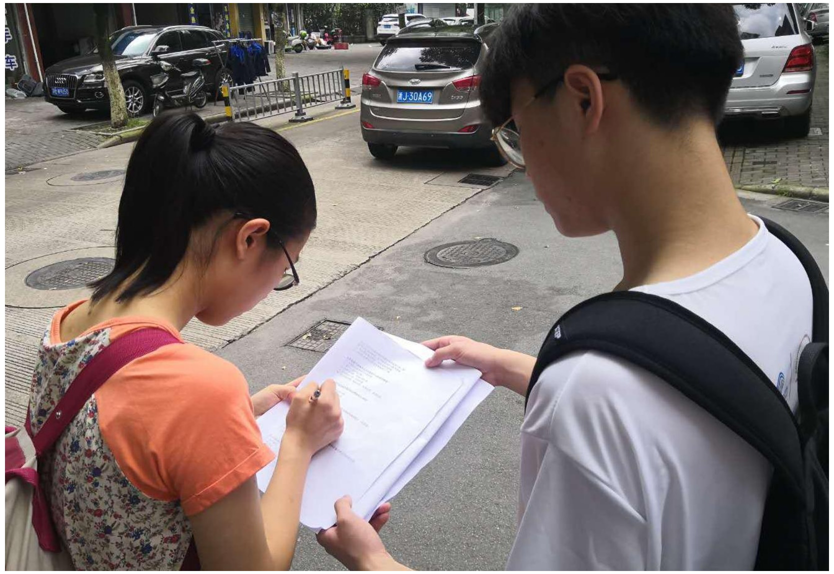

Fig 3. A team member is assisting the participant in filling out the questionnaire.

APPENDIX (FOR REVIEW ONLY)

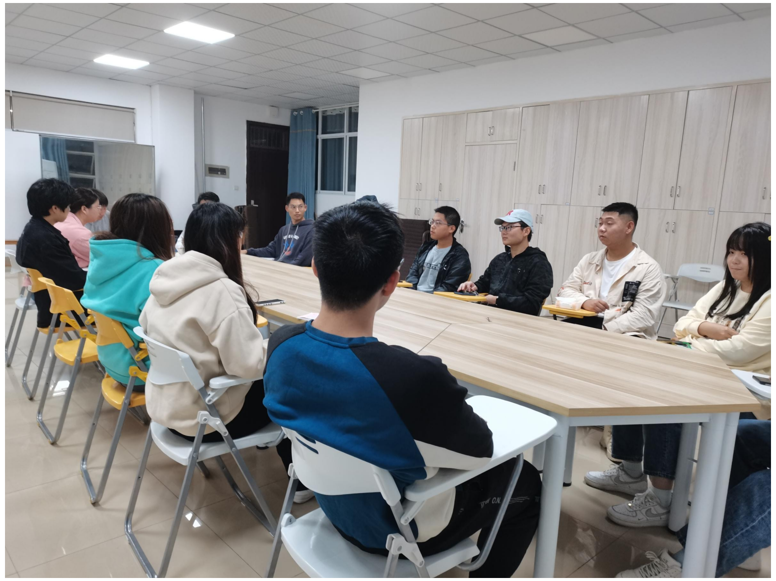

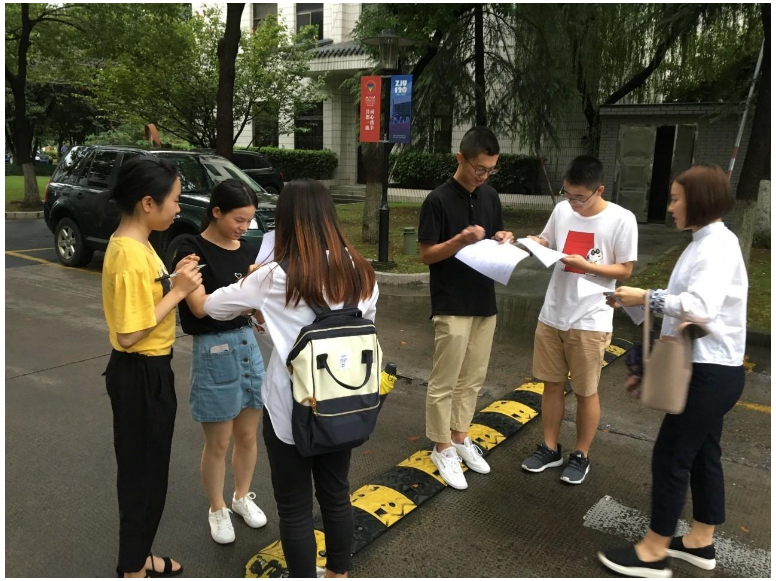

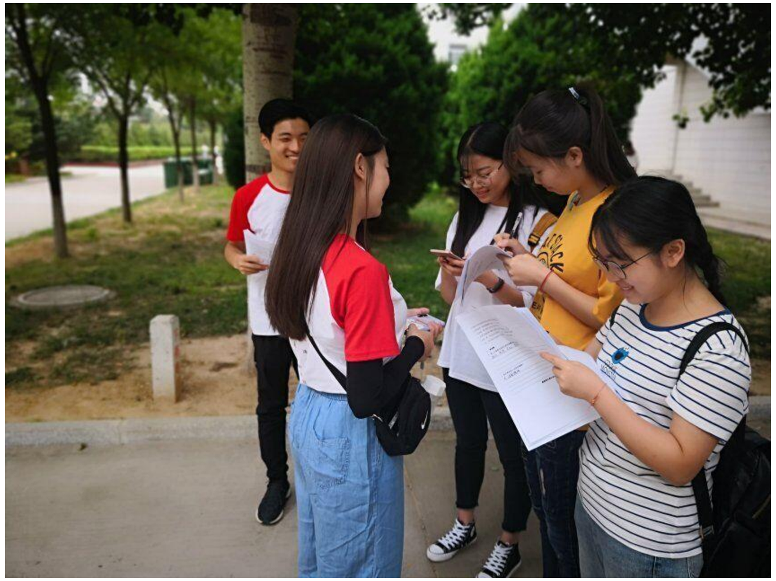

Fig 4. Team members are explaining the content of the questionnaire to the newly recruited participants.

Fig 5. Team members went into different neighborhoods to conduct offline questionnaire surveys.

Fig 6. Team members assisted participants in filling out the questionnaire and gave them rewards.

# APPENDIX (FOR REVIEW ONLY): Designed anthropomorphic packaging

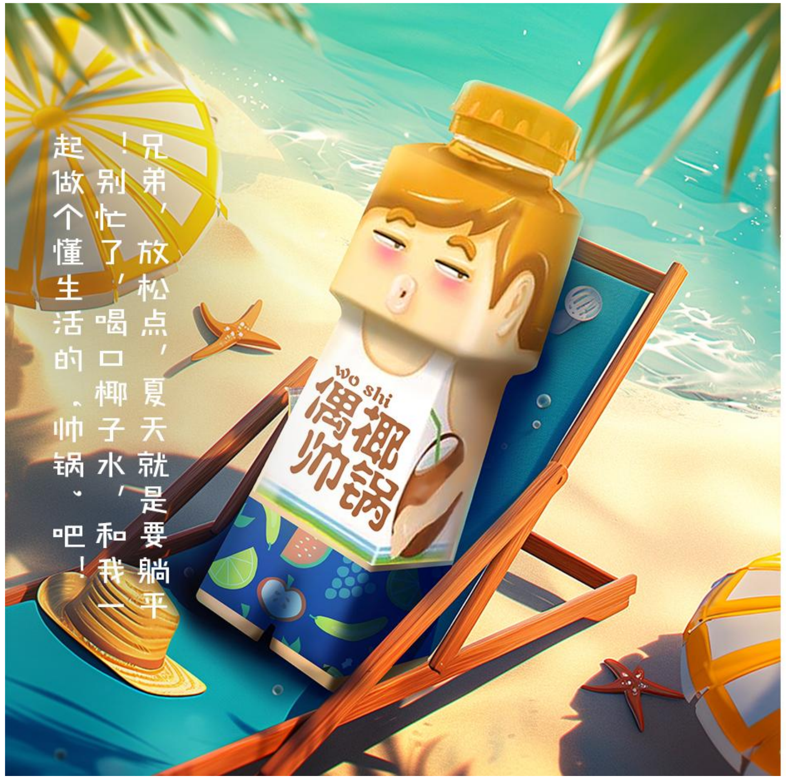


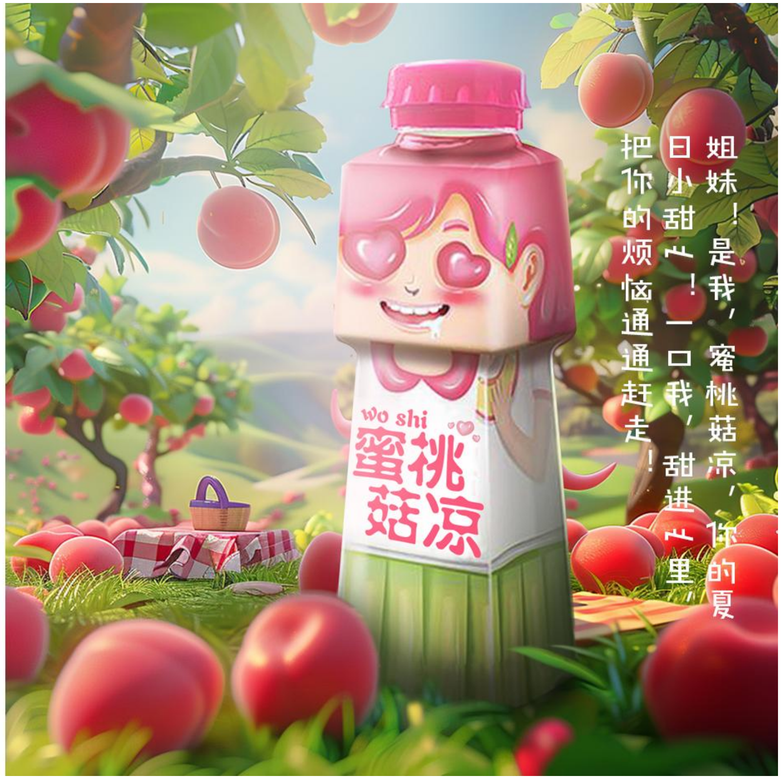


| Anthropomorphic Packaging Design Operation（visual anthropomorphism + verbal anthropomorphism） | |
|---|---|
| Coconut water beverage packaging appearance image design | "Hey bro, relax! Summer is all about enjoying the sunshine and the beach. Stop stressing, grab a sip of coconut water, and join me in being a cool guy who knows how to enjoy life!"<br>兄弟，放松点，夏天就是要躺平！别忙了，喝口椰子水，和我一起做个懂生活的‘帅锅’吧！ |
| Peach drink packaging appearance image design | "Hey sis! It’ s me, Peachy Little Fairy, your sweet summer bestie! One sip of me, and I’ ll sweeten your heart and chase all your worries away!"<br>姐妹！是我，蜜桃菇凉，你的夏日小甜心！一口我，甜进心里，把你的烦恼通通赶走！ |
