# Supplementary material for: Can anthropomorphic design in beverage packaging enhance impulse buying intention? Amazing visual and verbal cues!
Source: PLoS One. 2025 Jun 16;20(6):e0326186. doi: 10.1371/journal.pone.0326186 (PMC12169521; doi:10.1371/journal.pone.0326186)
Supplement: Attachment — Submitted filename: Response to Reviewers.pdf [file pone.0326186.s002.pdf]

Dear Reviewers,

Thank you very much for your thorough and constructive feedback on our manuscript titled "Can Anthropomorphic Design in Beverage Packaging Enhance Impulse Buying Intention? Amazing Visual and Verbal Cues!". This study systematically examines how anthropomorphic visual and verbal cues in beverage packaging influence consumers' impulse buying intentions. Grounded in the Stimulus-Organism-Response model, Social Presence Theory, and Empathy Theory, the research employs a consumer survey to validate the mediating roles of positive emotions, social presence, and empathy in the relationship between anthropomorphic design and impulse buying intentions. A comprehensive causal model was developed to explore these relationships. Based on the comments provided by the reviewers, significant modifications have been made to this study, including the establishment of a new model and the relationships between various variables. In addition, more relevant literature was consulted to support this study.

We sincerely appreciate the time and effort you have dedicated to reviewing our work. Your comments have been invaluable in helping us improve the quality and clarity of our paper. Below, we have addressed each of your comments and outlined the revisions we have made in response to your suggestions.

Sincerely,

Yuyang Tian
College of Creative Arts,University Technology Mara (Uitm), Shah Alam, Selangor, Malaysia

Respond to Reviewer:

Reviewer #1:

1. This research contains too many simple and isolated sentences, which might cause boredom and confusions to the readers. Below are just examples. The author should proofread the whole manuscript: "…This involves assigning human traits to non-human objects. Such design creates human-like appearances or behaviors. It aims to evoke emotional resonance in consumers. This strategy is widely used in marketing. It boosts product appeal and fosters consumer engagement…." "Trust is another key mechanism. It reduces perceived risk. Trust enhances product credibility and reliability. This increases consumer trust and purchase likelihood. Empathy is defined as the ability to understand and share others' emotions"

We thank you for pointing out the need for proofreading and improving sentence coherence. We have thoroughly revised the entire manuscript to ensure grammatical accuracy, clarity, and logical flow.

1 Introduction

In recent years, anthropomorphic design has emerged as a prominent marketing strategy~~.~~ ~~It is~~, increasingly utilized in the modern consumer market, particularly in product packaging. By attributing human-like characteristics to products, anthropomorphic design in beverage packaging can evoke emotional resonance among consumers~~. It enhances consumers'~~, enhancing their sense of identification with the product and ~~stimulates their purchase intentions [?]~~stimulating purchase intentions. In the Chinese market, attractive packaging is ~~the sole~~ a key design factor ranked among the top purchasing considerations~~[?]~~. Compared to other factors ~~,~~such as flavor innovation and pricing, optimizing packaging design appears to be a cost-effective and rapidly impactful marketing approach. For instance, the packaging of Intermarché's orange juice garnered 50 million media impressions within three hours of its launch~~. Within three weeks,~~, and sales surged by 4,600% ~~, and~~ within three weeks, with global in-store traffic ~~increased~~ increasing by 25% ~~due to its effective design strategy~~ (DIGITALING). ~~This anthropomorphic packaging not only created a sense of friendliness and fun among consumers but also significantly enhanced product competitiveness. It established a differentiation advantage in the market.~~ While the design strategy played a significant role, it is important to note that other factors such as brand awareness and promotional activities may have also contributed to this success. Similarly, VDA BANGKOK established TASTE&Co to implement innovative packaging designs~~. This initiative helped~~, helping small-scale producers in Thailand expand their businesses (Sohu). The global

Reviewer #1:

2. It is unclear about the context of this study. Is the impulse buying intention in a physical setting/m commerce/website/banner ads, etc.? This needs to be explained in more detailed.

We apologize for the lack of clarity regarding the study context. The research focuses on impulse buying intention in a physical retail setting, such as supermarkets or convenience stores. We have added a detailed explanation in the introduction to clarify this context and ensure readers understand the specific environment under investigation.

~~stimulates their purchase intentions [?]~~stimulating purchase intentions. In the Chinese market, attractive packaging is ~~the sole~~ a key design factor ranked among the top purchasing considerations ~~[?]~~. Compared to other factors ~~,~~ such as flavor innovation and pricing, optimizing packaging design appears to be a cost-effective and rapidly impactful marketing approach. For instance, the packaging of Intermarché's orange juice garnered 50 million media impressions within three hours of its launch~~. Within three weeks,~~ , and sales surged by 4,600% ~~, and~~ within three weeks, with global in-store traffic ~~increased~~ increasing by 25% ~~due to its effective design strategy~~ (DIGITALING). ~~This anthropomorphic packaging not only created a sense of friendliness and fun among consumers but also significantly enhanced product competitiveness. It established a differentiation advantage in the market.~~ While the design strategy played a significant role, it is important to note that other factors such as brand awareness and promotional activities may have also contributed to this success. Similarly, VDA BANGKOK established TASTE&Co to implement innovative packaging designs~~. This initiative helped~~ , helping small-scale producers in Thailand expand their businesses (Sohu). The global beverage packaging market was valued at ~~$138.72 billion in 2022. It is expected to grow to~~ $138.72billionin2022andisexpectedtogrowto204.53 billion by 2030, with a compound annual growth rate of 5.1% from 2023 to 2030 (DATA BRIDGE). This growth trend underscores the importance of innovative packaging design in driving market expansion.

Reviewer #1:

3. Line 61: anthropomorphic packaging was mentioned, however, it was not explicitly explained what is meant by anthropomorphic packaging. In addition, there are many weak supporting evidence. For example, it was stated that "sales surged by 4,600%", is it merely due to anthropomorphic packaging? Are there any other factors, e.g. high brand awareness due to investment in ads, or promotion?. If the "surge" is in conjunction with many other factors, then these evidence are not strong enough to conclude that "These data suggest that anthropomorphic design in packaging significantly enhances consumers' emotional identification and purchase intentions"

Thank you for highlighting the need for a clearer definition and stronger evidence. We have explicitly defined anthropomorphic packaging in the introduction (Lines 95–99) as "the assignment of human traits, such as facial features, emotions, or language, to beverage packaging to evoke emotional connections with consumers." Additionally, we have revised the discussion of the 4,600% sales surge (Lines 66–72) to acknowledge that other factors, such as advertising and promotions, may have contributed to the increase. We now present this example as a supplementary illustration rather than conclusive evidence.

> Anthropomorphic design is a strategy that evokes emotional resonance in consumers by assigning human traits to products [3]. These traits include anthropomorphic elements in appearance, such as facial expressions and body shapes~~. They also involve~~, as well as emotional and personality expressions conveyed through verbal communication. Despite its widespread application in marketing, the influence of anthropomorphic design on consumer purchase behavior through psychological mechanisms such as ~~emotional response~~ affective responses and cognitive evaluation remains underexplored

> impactful marketing approach. For instance, the packaging of Intermarché's orange juice garnered 50 million media impressions within three hours of its launch~~. Within three weeks,~~, and sales surged by 4,600% ~~, and~~ within three weeks, with global in-store traffic ~~increased~~ increasing by 25% ~~due to its effective design strategy~~ (DIGITALING). ~~This anthropomorphic packaging not only created a sense of friendliness and fun among consumers but also significantly enhanced product competitiveness. It established a differentiation advantage in the market.~~ While the design strategy played a significant role, it is important to note that other factors such as brand awareness and promotional activities may have also contributed to this success. Similarly, VDA BANGKOK established TASTE&Co to implement innovative packaging designs~~. This initiative helped~~, helping small-scale producers in Thailand expand their businesses (Sohu). The global

Reviewer #1:

4. Line 74: There are many weak arguments, for example "whether anthropomorphic language styles influence consumer behavior through psychological mechanisms such as social presence, positive emotions, and empathy remains insufficiently explored." Actually, there have been many research on anthropomorphic language styles and social presence. There are just a few references in the introduction.

We appreciate your observation regarding the weak arguments on anthropomorphic language styles. We have expanded the literature review (Lines 82–90) to include more recent and relevant studies on anthropomorphic language, social presence, and their psychological mechanisms. Additional

references have been added to strengthen the theoretical foundation of our research.

morphic designs remain underexplored. Most existing studies focus on the impact of visual designs on consumers, such as cartoon patterns and anthropomorphic appearances~~.~~ ~~There is~~ , with limited attention given to the role of verbal cues in anthropomorphic beverage packaging designs. For example, whether anthropomorphic language styles influence consumer behavior through psychological mechanisms such as social presence, positive emotions, and empathy remains insufficiently explored ~~. Additionally, consumers' responses to anthropomorphic designs may vary across different contexts. Consumers with higher levels of loneliness may be more susceptible to anthropomorphic designs. However, this hypothesis has yet to be validated.~~ [2]. Therefore, further research is needed to examine the roles of visual and verbal cues in anthropomorphic design ~~. It is crucial to~~ and explore how these cues affect consumer behavior through complex psychological mediation mechanisms. ~~Moreover, investigating the moderating role of loneliness in this process holds significant academic and practical value.~~

Reviewer #1:

5. Line 122: The author stated that "Anthropomorphic beverage packaging, which assigns human traits to products…". However, the first hyperlink (DIGITALING) in line 61 shows photos of fruits (e.g. banana and strawberry) and animals (e.g. cat). The definition is not actually consistent to examples given. This will cause confusions to readers.

We apologize for the inconsistency between the definition and examples provided. We have revised the examples in Lines 61–65 to align with our definition of anthropomorphic packaging, focusing on human-like traits (e.g., facial features or emotional expressions) rather than fruits or animals. This change ensures consistency and avoids confusion.

Reviewer #1:

6. Line 164: The author has immediately switched from Concept of Impulse Buying Intention to SOR. It is important to create a reading flow for readers. In addition, the author needs to justify how SOR framework is applicable in the context of Impulse Buying Intention. Furthermore, SOR model should be explained in greater details.

Thank you for pointing out the abrupt transition to the SOR (Stimulus-Organism-Response) framework. We have added a bridging paragraph (Lines 164) to explain the connection between impulse buying intention and the SOR model. Additionally, we have provided a more detailed

explanation of the SOR framework (Lines 192–211) and justified its applicability to our study context.

Organism-Response (S-O-R) model provides a ~~theoretical framework to explain~~ robust theoretical framework for understanding the formation of impulse purchase intentions. ~~In~~ According to this model, external stimuli (S) influence an individual's internal states (O), which in turn drive behavioral responses (R). In the context of this study, anthropomorphic beverage packaging ~~acts as an~~ serves as the external stimulus, utilizing visual and verbal cues to ~~affect consumer emotions and cognition~~evoke emotional and cognitive responses in consumers. These internal responses, ~~in turn, influence~~ such as heightened positive emotions or a sense of social connection, then lead to increased impulse purchase intentions. The S-O-R model is particularly applicable to this research because it captures the dynamic interplay between environmental stimuli (e.g., anthropomorphic packaging), psychological processes (e.g., emotional resonance

and trust), and behavioral outcomes (e.g., impulse buying). For example, Fang et al. (2022) [19] argue that weakened self-control, as ~~per~~ explained by self-regulation theory, increases the likelihood of impulse buying. Emotional induction theory further suggests that positive emotions reduce self-control, ~~increasing impulse purchases. Trust enhances consumers ' sense of safety and reduces decision uncertainty, encouraging quicker purchases [?]. Moreover, loneliness amplifies the desire for social connection. Anthropomorphic designs mimic interpersonal interactions, fulfilling social needs and promoting impulse purchases under such conditions.~~ making consumers more susceptible to impulsive decisions.

Reviewer #1:

7. In regards with questionnaires used in this research, all questions for Social presence are about human-likeness, which is the same as anthropomorphism. Therefore, it is very difficult to come up with a sound analysis. In addition, in table 1, sources of questionnaire measurement items were provided, however, it is unclear which one the author has adapted because the author has provided at least 2 sources for 1 construct. Take a look at reference 64, 5 items for social presence are much different from the items used in this research. This might be due to poor application of back translating technique, leading to inaccurate analyses and findings.

We acknowledge the issues with the questionnaire design and measurement items. To address this, we have: Revised the social presence questions to better distinguish between anthropomorphism and social presence. Clarified the sources of measurement items in Table 1, specifying which items were adapted from each reference. Conducted a rigorous back-translation process to ensure the accuracy of the translated items.

58. Gefen, D., & Straub, D. (2003). Managing user trust in B2C e-services. *e-Service*, *2*(2), 7–24.

59. Lim, X.-J., Cheah, J.-H., Ng, S. I., Basha, N. K., & Soutar, G. (2021). The effects of anthropomorphism presence and the marketing mix on retail app continuance use intention. *Technological Forecasting and Social Change, 168*, 120763.

60. Gao, W., Liu, Z., & Li, J. (2017). How does social presence influence SNS addiction? A belongingness theory perspective. *Computers in Human Behavior, 77*, 347–355.

Reviewer #1:

8. Line 454: it is unclear if the author conducted an experimental research, or a Covariance-Based Structural Equation Modeling.

We apologize for the lack of clarity regarding the research methodology. We have explicitly stated in the methodology section (Lines 691) that this study employs Covariance-Based Structural Equation Modeling (CB-SEM) to analyze the data. A detailed explanation of the CB-SEM approach has also been added.

4.6 Structural Equation Model Path Analysis

~~Structural equation modeling (SEM) was used to test the path coefficients of the hypothesized model . The analysis examined the direct effects of beverage packaging anthropomorphic design (visual and verbal cues) on impulse buying intention and its mechanisms. Table 8 indicates that most hypotheses were supported. Anthropomorphic design (visual cues) positively influences impulse buying intention. The path coefficient is 0.129, with a standard error of 0.057, a critical ratio (C.R.) of 2.257, and a p-value of 0.024, confirming this hypothesis. Similarly, anthropomorphic design (verbal cues) positively affects impulse buying intention, with a path coefficient of 0.252, a standard error of 0.070, a C.R. of 3.593, and a p-value < 0.001, indicating a stronger effect. Visual cues moderately enhance positive emotions (path coefficient = 0.134, standard error = 0.067, C.R. = 1.998, p-value = 0.046). They also strongly enhance~~ This study employed a structural equation model to analyze each path coefficient in detail. The model fit was good. The theoretical constructs and empirical data were highly consistent. The results (see Table 8) show that visual cues in packaging design significantly enhanced social presence (~~path coefficient~~ β = ~~0.512, standard error~~ 0.506, SE = 0.061, C.R. = [illegible]

Reviewer #1:
9. Marker variable test for common method bias check was missing.

Thank you for noting the omission of the marker variable test. We have now included this test in the methodology section (Lines 410–415) and confirmed that common method bias is not a significant concern in our study.

~~This study validated multiple hypothesized relationships using exploratory factor analysis, confirmatory factor analysis, and structural equation modeling (SEM~~The empirical results of this study generally align with theoretical expectations (see Figure 3). The ~~results demonstrate strong consistency between the theoretical framework and empirical data, highlighting the connection between theory and practice. Figure 3 shows that both visual and verbal anthropomorphic designs in beverage packaging significantly affect~~ findings reveal some subtle differences. Visual cues and verbal cues both effectively enhance consumers' ~~impulse buying intentions. Visual anthropomorphic design promotes impulse buying by enhancing social presenceand positive emotions. Visual elements, such as anthropomorphic facial expressions and imagery, effectively evoke consumers'~~ sense of social presence, which supports social presence theory. Products simulate interpersonal interaction to provide a context for emotional experiences and ~~social connections. These findings align with the S-O-R model and social presence theory, showing how external stimuli influence behavior by altering consumers' internal states. However, visual anthropomorphic design did not significantly affect empathy, likely due to the limited capacity of visual cues to evoke deep emotional resonance. In contrast, verbal anthropomorphic design directly influences impulse buying intentions and indirectly impacts purchase behavior by enhancing social presence , positive emotions,~~ subsequent behavior. Although theory predicts that social presence will trigger empathy, the revised empirical results confirm a strong direct relationship between them, indicating that a heightened sense of social presence is indeed a critical antecedent to evoking empathy. In addition, positive emotions are influenced by both social presence and

Reviewer #1:
10. Headings were not appropriately deployed, for example, Line 847

We have revised the headings throughout the manuscript to ensure they are appropriately deployed and consistent with journal guidelines. For example, the heading in Line 847 has been rephrased to better reflect the content of the section.

Questionnaire Questions

References

1. Yuting T, Mohamed F. Examining Consumer Perception and Purchase Intention of Gift Packaging: A Case Study of Chinese Consumers on Green Tea Packaging. International Journal of Academic Research in Business and Social Sciences. 2023;13(2).

Questionnaire Information

Anthropomorp Design (Visual Cues)	1 – The product (packaging) looks like a person.<br>2 – The product (packaging) has a human-like expression.<br>3 – The product (packaging) has a human-like appearance.<br>4 – The product (packaging) has come alive.
Anthropomorp Design (Verbal Cues)	1 – The verbal style of this product (packaging) is natural.<br>2 – The verbal style of this product (packaging) is humanlike.<br>3 – The verbal style of this product (packaging) is conscious.<br>4 – The verbal style of this product (packaging) is elegant.
Impulse Buying Intention	1 – When I saw the product (packaging), I had a strong desire to buy it.<br>2 – When I saw the product (packaging), I wanted to have it immediately.<br>3 – When I saw the product (packaging), I felt that the product was what I wanted.<br>4 – When I saw the product (packaging), I wanted to buy it even though it was not what I had planned to buy before.
Positive Emotions (Pleasure & Arousal)	1 – When I saw the product (packaging), I felt happy.<br>2 – When I saw the product (packaging), I felt pleasure.<br>3 – When I saw the product (packaging), I felt hopeful.<br>4 – When I saw the product (packaging), I felt satisfied.<br>5 – When I saw the product (packaging), I felt active.<br>6 – When I saw the product (packaging), I felt excited.
Social Presence	1 – There is a sense of human contact with this product (packaging).<br>2 – This product (packaging) has a personalized touch.<br>3 – There is a human-like warmth associated with this product (packaging).<br>4 – There is a sense of human sensitivity associated with this product (packaging).
Empathy	1 – While watching the product (packaging), I can feel the packaging's emotions.<br>2 – While watching the product (packaging), I experienced many of the same feelings that the packaging portrayed.<br>3 – While watching the product (packaging), I can understand what the packaging is going through.<br>4 – While watching the product (packaging), I felt like I was the packaging.

References

Reviewer #1:

11. Control variables in the research model and analysis were missing.

We appreciate your suggestion to include control variables. We have added control variables such as age, gender, and income level to the research model and analysis. This adjustment ensures a more robust examination of the factors influencing impulse buying intention.

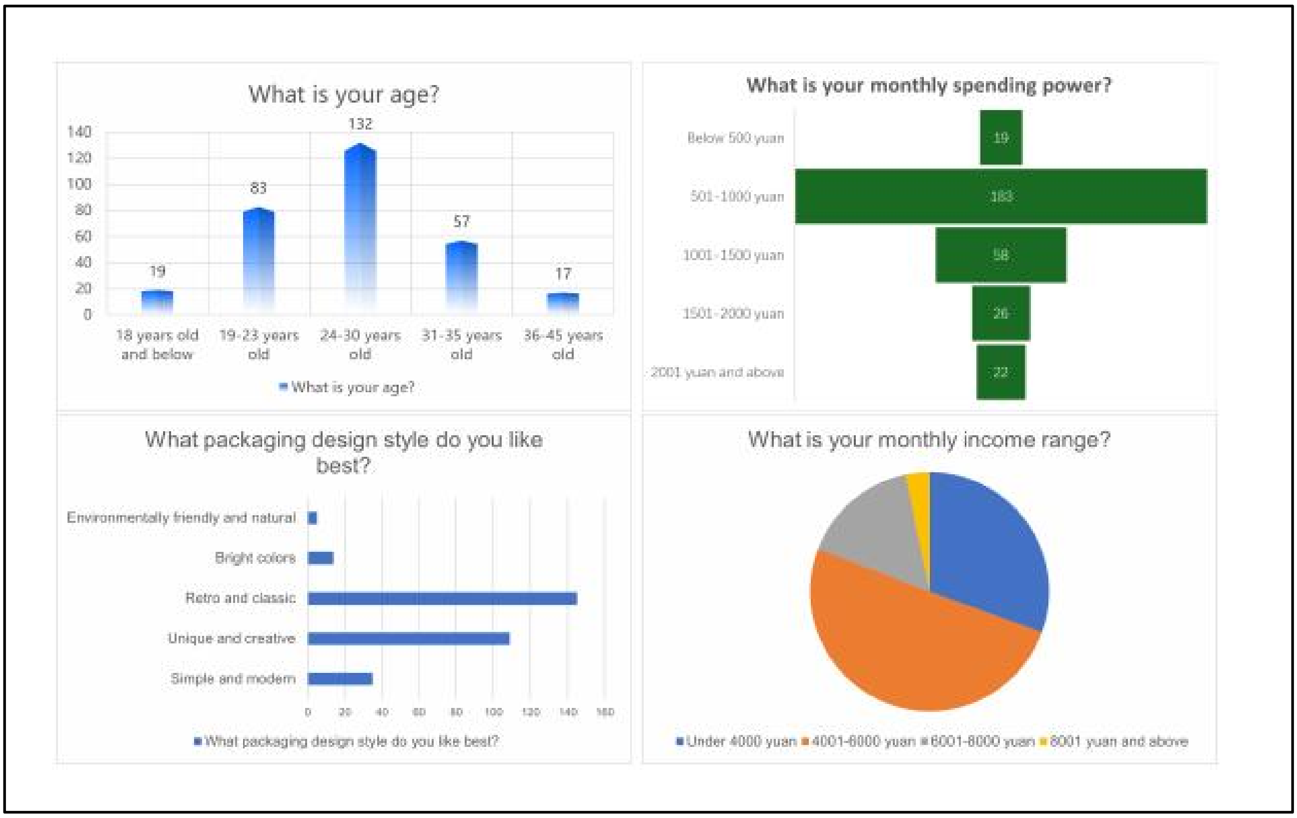

Reviewer #2:

1. In my opinion, one of the most fundamental weaknesses in this manuscript is the lack of coherence in the theoretical model. While the authors discussed each path between variables and cite theories to justify individual relationships, the model feels fragmented rather than conceptually unified. It appears as though the mediators (social presence, positive emotions, and empathy) were selected based on convenience rather than a systematic theoretical rationale. This raises a critical concern: Why were these three specific mediators chosen over other potential psychological mechanisms? Currently, the model lacks theoretical integrity such that it feels piecemeal rather than cohesive. The authors cited theoretical justifications for individual pathways, but they did not justify why these mediators should be examined together within the same framework. This suggests that the authors may not have considered how these mediators interact or why they are the most important psychological mechanisms in this context.

As currently presented, positive emotions, social presence, and empathy are conceptually distinct and do not seem to align under a single unifying theme. Therefore, I would suggest the authors to find a way to clearly demonstrate why these variables collectively form the most appropriate psychological mechanisms through which anthropomorphic design influences impulse buying intention. In other words, the authors are suggested to integrate these variables into a coherent framework and explain it clearly in the literature review. For example, the authors may think of categorizing mediators into one or more broader constructs that can align with each other under a single theme. Alternatively, if the authors want to maintain them as distinct variables, they should explain how these three mediators complement

each other rather than operating in isolation.

We sincerely thank you for identifying the lack of coherence in our original theoretical model. We agree that the mediators (social presence, positive emotions, and empathy) were not sufficiently integrated into a unified framework, which made the model appear fragmented. To address this critical concern, we have revised the theoretical model and provided a more systematic rationale for the selection and integration of these mediators.

3 Hypotheses

3.1 Conceptual model

The conceptual model is shown as Fig. 1:

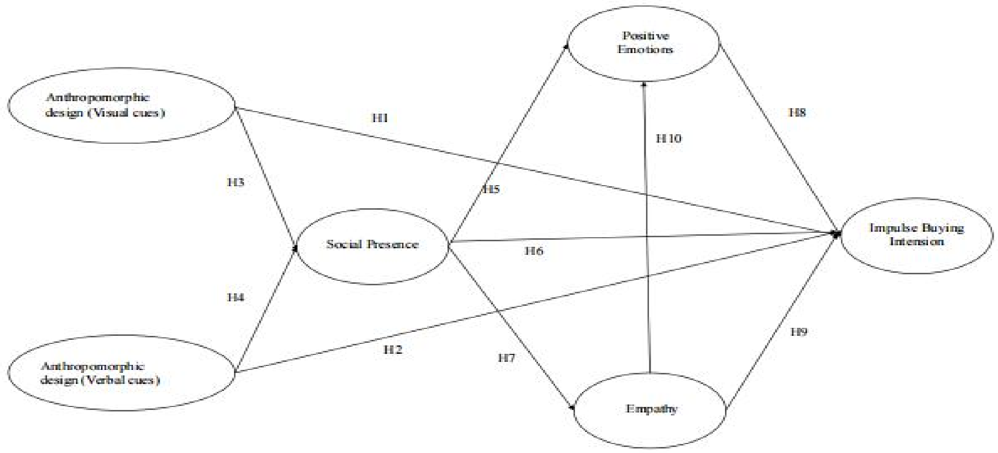

Fig 1. Research model.

2.2 ~~Anthropomorphic Design~~ Affective Response Theory~~and Psycholog~~ ~~Mechanisms~~

~~Anthropomorphism is a psychological process. It is driven by three core human needs: understanding the world, forming connections, and maintaining a sense of belonging [?]. Vrtana and Križanová (2023) [?] explain anthropomorphic design. This involves assigning human traits to non-human objects. Such design creates~~ Affective Response Theory In Affective Response Theory (ART), consumers' emotional reactions to external stimuli play a crucial role in shaping their behavioral responses [20]. Affective responses are immediate and automatic reactions to stimuli, which subsequently influence cognitive evaluations and decision-making processes [21]. ART is widely applied in marketing and consumer behavior research to explain how emotional engagement with products, advertisements, or packaging designs drives consumer actions, such as purchase intentions and brand loyalty [22]. In the context of anthropomorphic design, ART provides a robust framework for understanding how human-like ~~appearances or behaviors. It aims to evoke emotional resonance in consumers. This strategy is widely used in marketing . It boosts product appeal and fosters consumer engagement. Theoretical frameworks supporting it include anthropomorphism theory, social presence theory, and the S-O-R~~

Reviewer #2:

2. In the current version of this paper, moreover, loneliness was treated as a moderator without adequate theoretical grounding. The choice of loneliness as a moderator lacks a compelling justification beyond speculative reasoning. The paper assumes that lonely consumers will respond more strongly to anthropomorphic packaging, but it does not explain why loneliness is the most relevant moderator over other psychological states. Ideally, a good and appropriate moderator should not deviate dramatically from the focal point of the main model/research context. Conceptually, it should be closely linked with IVs and (or) outcome variables. However, I cannot see why loneliness fits in this paper. Moreover, the proposed moderating effect of loneliness (i.e. the effects of anthropomorphic design will be stronger for high-loneliness consumers) is too obvious. Please do not get me wrong here – While intuitive insights can be valuable, readers may question whether this research is actually uncovering new consumer insights or simply confirming common sense. If a moderator's effect is overly self-evident, it diminishes the contribution of the study because it does not challenge or
refine existing theoretical perspectives.

Therefore, if the authors wish to retain loneliness as the moderator, I suggest (1) Explaining clearly why loneliness is the most relevant psychological state/moderator that need to be considered in this research; (2) refining the argument to introduce theoretical complexity in hypotheses development; and (3) Considering testing an additional moderator to strengthen the study's robustness.

We greatly appreciate your observation regarding the insufficient theoretical grounding for loneliness as a moderator. Upon careful consideration, we agree that the inclusion of loneliness lacked a compelling justification and that its moderating effect was overly intuitive, which diminished the theoretical contribution of the study. Revisions Made:

Removal of Loneliness: We have decided to remove loneliness as a moderator from the theoretical model. This decision was made to ensure that the study focuses on more robust and theoretically relevant mechanisms.

Strengthening the Core Model: By removing loneliness, we have redirected our focus to refining and strengthening the core relationships in the model, particularly the mediating roles of positive emotions, social presence, and empathy. This allows us to provide a more in-depth analysis of the primary psychological mechanisms through which anthropomorphic packaging influences impulse buying intention.

3 Hypotheses

3.1 Conceptual model

The conceptual model is shown as Fig. 1:

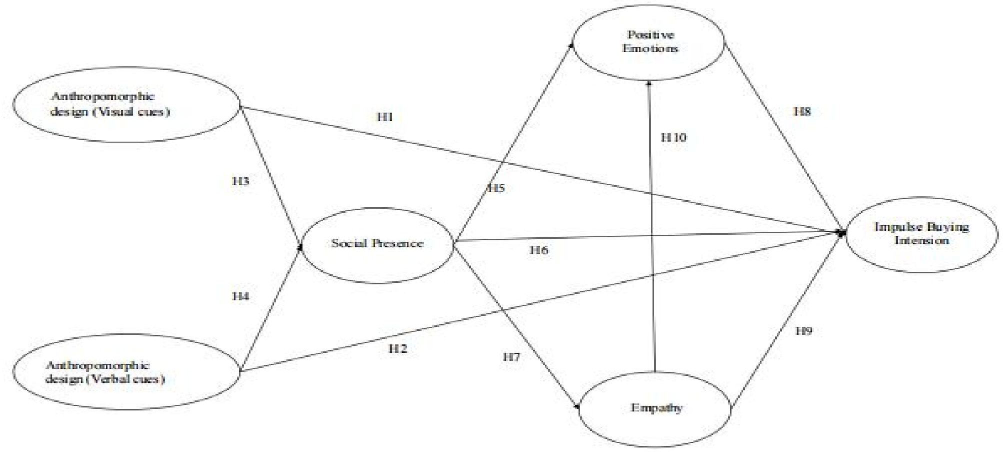

Fig 1. Research model.

~~H4: Anthropomorphic design in beverage packaging (visual cues)~~ H5: Social presence positively influences positive emotions.

H6: Social presence positively influences impulse buying intention.

H7: ~~Anthropomorphic design in beverage packaging (verbal cues) positively influences positive emotions~~Social presence positively influences empathy.

~~beverage packaging evoke state empathy, enhancing consumers'~~ make impulse purchases [38]. In addition, in the SOR model, stimuli such as product packaging or store environment can elicit affective responses (positive emotions) from consumers, which in turn drive behavioral responses (impulse buying) [39]. Positive emotions play a mediating role between external stimuli and impulsive buying behavior [40]. Therefore, anthropomorphic packaging can evoke consumer reactions and lead to the purchase of products. Rook et al. (2022) found that positive emotions such as excitement and happiness significantly increase the likelihood of impulse buying [41]. Their research emphasizes that consumers who experience positive emotions are more likely to take action on spontaneous desires without deep consideration. Herabadi explored the role of emotional states in impulse buying and found that positive emotions significantly increased impulse buying tendencies. They believe that positive emotions can lower consumers' self-control and make them more likely to make impulsive decisions. Zhang et al. studied the impact of positive emotions on impulse buying in online shopping contexts. Their research findings indicate that positive emotions, such as happiness and satisfaction, significantly enhance impulse buying intentions ~~and amplifying positive emotions. Therefore~~by increasing consumers' perceived enjoyment and reducing their hesitation. Hence, the following ~~hypotheses are proposed:~~

~~H5: Anthropomorphic design in beverage packaging (visual cues) positively influences empathy~~hypothesis is constructed.

H8: ~~Anthropomorphic design in beverage packaging (verbal cues) positively influences empathy~~Positive Emotions positively influences impulse buying intention.

Reviewer #2:

3. The study surveyed 308 beverage consumers in Shenzhen, yet the authors do not provide any justification for why Shenzhen is an appropriate market for this study. This is a critical omission because market characteristics (e.g., consumer demographics, cultural tendencies, purchasing behaviours) can significantly influence the results. Without this justification, it is unclear whether the findings can be generalized to other markets or if Shenzhen represents a unique consumer group. Additionally, the manuscript did not specify how the sample was recruited. Was it through random sampling, convenience sampling, or quota sampling? The lack of transparency in sampling methodology raises concerns about potential selection bias, which could impact the study's validity.

The authors also need to justify why a sample size of 308 is appropriate for this study. Given that the proposed model consists of seven constructs, a larger sample would typically be expected to ensure adequate statistical power and model stability in SEM. It would be useful for the authors to conduct and report a power analysis to determine whether 308 responses provide sufficient power to detect meaningful effects. Lastly, the manuscript misuses the term "experimental results" when describing its findings. Since the study is purely survey-based without an experimental design (i.e. there are no control vs. treatment groups), the term "experimental" is misleading and should be replaced with more appropriate terminology such as "survey findings" or "empirical results" to avoid confusion.

We sincerely thank you for pointing out the lack of justification for selecting Shenzhen as the study market. We agree that market characteristics, such as consumer demographics and cultural tendencies, can significantly influence the results, and we have now provided a detailed rationale for our choice of Shenzhen.

Shenzhen is a major metropolitan city in China with a diverse and dynamic consumer base, making it an ideal market for studying impulse buying behavior. Its residents are known for their high purchasing power, openness to innovative product designs, and responsiveness to marketing stimuli, which align well with the objectives of this study. Additionally, Shenzhen's status as a hub for beverage innovation and its competitive retail environment provide a rich context for examining the effects of anthropomorphic packaging.

Due to the modification of the research model, the methodology section has also been adjusted accordingly (Lines 647-998). We thank you for pointing out the inappropriate use of the term "experimental results." We agree that this terminology is misleading, as the study is survey-based and does not involve an experimental design.

~~design in beverage packaging (visual and verbal cues) and impulse buying intentions.~~

~~H13a: Loneliness significantly moderates the relationship between beverage packaging anthropomorphic design (visual cues) and~~ H9: Empathy positively influences impulse buying intention.

~~H13b: Loneliness significantly moderates the relationship between beverage packaging anthropomorphic design (verbal cues) and impulse buying intention~~H10: Empathy positively influences positive emotions.

4 Empirical Results

4.1 Questionnaire Source

The questionnaire for this study aims to investigate the effects of anthropomorphic beverage packaging design on impulse buying intentions and its underlying mechanisms. The questionnaire is divided into ~~four~~ third sections: design elements, consumer emotional responses, individual psychological traits, and purchase behavior. The first section covers Anthropomorphic Design, specifically focusing on visual and verbal cues. The second section addresses Positive Emotions, Social Presence, and Empathy. The third

Reviewer #2:

4. While the methodological section is generally solid, the authors failed to test for common method variance, which is a significant concern in survey-based research. To address this, the authors need to include a formal test for CMV, using Marker variable / ULMC / other methods. Please note that Harman’s single-factor test is insufficient and should not be used.

We sincerely thank you for highlighting the need to formally test for common method variance (CMV) in our survey-based research. We acknowledge that CMV is a significant concern in studies relying on self-reported data, and we have taken steps to address this issue in the revised manuscript.

Formal CMV Test: As recommended, we have conducted a formal test for CMV using the Unmeasured Latent Method Construct (ULMC) approach, which is widely regarded as a robust method for detecting and controlling CMV. This approach involves adding a latent method factor to the structural equation model (SEM) and comparing the variance explained by the substantive constructs with and without the method factor. The results indicate that CMV does not significantly bias our findings, as the variance explained by the method factor was minimal (less than 10%).

Reporting the Results: We have added a detailed description of the ULMC procedure and its results to the methodology section (Lines647-781). This includes the steps taken to implement the test, the statistical outcomes, and an interpretation of the findings.

Avoidance of Harman's Single-Factor Test: In line with your recommendation, we have not used Harman's single-factor test, as it is considered insufficient for detecting CMV. Instead, we have relied on the more rigorous ULMC approach to ensure the validity of our results.

Reviewer #2:

5. The authors did not mention whether control variables were included in the analysis, yet several key consumer characteristics are likely to influence impulse buying behaviours. Specifically, brand familiarity, product experiences, and prior purchasing behaviours are known to have strong effects on consumer decision-making. Without controlling for these factors, the authors risked overestimating the effect of anthropomorphic design because the observed relationships could be partially explained by consumers' pre-existing attitudes / experiences. If control variables were included, the authors need to explicitly state which ones were used, how they were measured, and how they influenced the results. If no control variables were included, the authors must provide a strong justification for why they were omitted and acknowledge this as a limitation.

We appreciate your suggestion to include control variables. We have added control variables such as age, gender, and income level to the research model and analysis. This adjustment ensures a more robust examination of the factors influencing impulse buying intention.

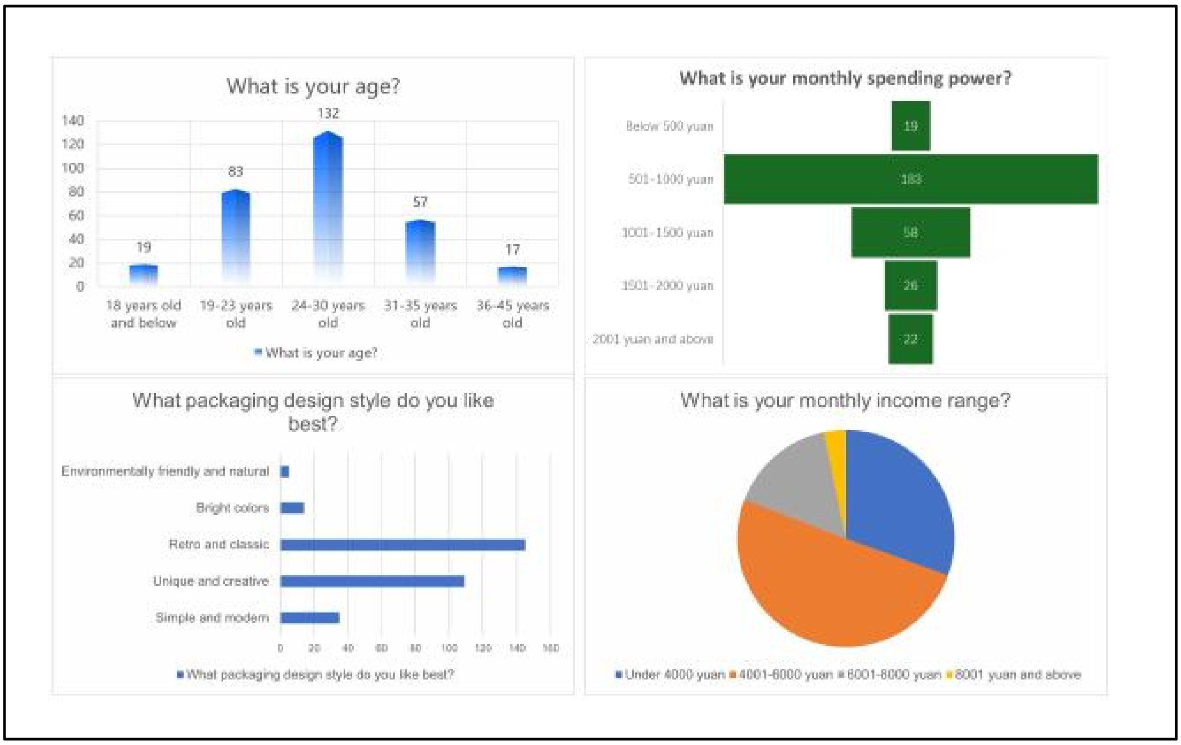

Reviewer #2:

6. I found that many sections (especially in Theoretical development, Hypotheses, and Results discussion) repeated the same points multiple times. Please streamline the manuscript to remove redundancy. It also seems that the authors put too much emphasis on discussing concepts and theories (e.g., trust, Self-congruity theory, etc.) that irrelevant to the focal points of this research. If I were wrong about this, then please clearly explain how they related to this research. Otherwise, please reduce these discussions which may distract readers. In my opinion, the implications are generic and lack depth. The study concludes that anthropomorphic design can enhance impulse buying, but it fails to translate these findings into concrete marketing strategies that beverage companies can use. Please find a way to strengthen the practical implications, focusing on how beverage companies should specifically implement anthropomorphic design strategies.

We sincerely thank you for pointing out the issues of redundancy, overemphasis on less relevant theories, and the lack of depth in practical implications. We have carefully revised the manuscript to address these concerns.

5 Implications

5.1 Theoretical Implications

This study ~~uses~~ employs empirical analysis to ~~examine~~ explore how visual and verbal cues in anthropomorphic beverage packaging design influence consumers' impulse buying intentions. It contributes to ~~theoretical research in~~ the theoretical understanding of anthropomorphic design and consumer behavior by validating the applicability of the Stimulus-Organism-Response (S-O-R) model. The ~~study shows~~ findings demonstrate that external stimuli (anthropomorphic design) influence ~~behavior~~ behavioral responses (impulse buying intention) by shaping internal psychological states~~(~~, including positive emotions, social presence, and empathy~~)~~. This mechanism is empirically supported [69]. The study ~~highlights~~ underscores the distinct roles of visual and verbal cues in anthropomorphic design. Visual cues primarily enhance positive emotions and social presence, ~~whereas~~ while verbal cues evoke stronger empathetic responses. These findings provide deeper insights into the mechanisms underlying different types of cues in anthropomorphic design. They also ~~support~~ align with Lim et al. (2021)'s [70] cognitive appraisal theory, ~~suggesting~~ which posits that verbal information induces deeper cognitive processing and attitude changes. Furthermore, the study ~~emphasizes the moderating role of loneliness in anthropomorphic design. Consumers experiencing higher levels of loneliness respond more strongly to the positive emotions and impulse buying intentions evoked by anthropomorphic design. This finding aligns with Barnuevo et al. (2023)'s [?] theory of loneliness, demonstrating that psychological states shape consumers' responses to external stimuli. Additionally, the study shows that~~ reveals that visual anthropomorphic design does not significantly affect empathy~~. This suggests~~, suggesting that verbal cues are more effective in eliciting deep emotional resonance~~,~~

5.2 Practical Implications

This study ~~offers~~ provides valuable insights for businesses in beverage packaging design and marketing strategies. First, verbal anthropomorphic design plays a more ~~prominent~~ significant role than visual design in stimulating consumers' impulse buying intentions. It evokes positive emotions, enhances social presence, and triggers empathy. This finding aligns with Huang et al. (2023) [71] cognitive appraisal theory, ~~suggesting~~ which suggests that detailed verbal information induces deeper cognitive processing and attitude changes. Therefore, businesses should incorporate emotional language, humor, and personalized expressions into packaging ~~. This approach conveys~~ to convey product emotions and personality, thereby influencing consumer decisions. Since visual design does not significantly impact empathy, combining visual and verbal elements can create multidimensional and vivid product images. For example, pairing anthropomorphic cartoon characters with emotional statements on packaging can address the limitations of visual cues and enhance marketing effectiveness [72]. Additionally, businesses should ~~target consumers experiencing higher levels of loneliness. The study reveals that loneliness moderates the relationship between anthropomorphic design and impulse buying intentions. Higher levels of loneliness amplify the effects of anthropomorphic design. This finding aligns with Dang and Liu (2023) [?] theory of loneliness, indicating that businesses should address consumers' emotional needs in market segmentation. Companies can identify high-loneliness consumer groups through market research and data analysis. They can design and promote anthropomorphic packaging to fulfill these consumers' social and emotional needs, boosting brand loyalty and purchase intentions. Moreover, businesses should utilize social~~ leverage social media's interactivity and reach to ~~enhance~~ amplify the impact of anthropomorphic design. By ~~combining~~ integrating online and offline strategies, ~~businesses~~ companies can share anthropomorphic product images and stories to enhance consumer engagement and build a sense of community. Finally, companies should continuously monitor consumer behavior and market trends to dynamically adjust their strategies. They should innovate anthropomorphic ~~design to address~~ designs to cater to diverse cultural needs, ensuring effectiveness and competitiveness in the global market.

6 Conclusion

This study ~~investigates~~ investigated how anthropomorphic design in beverage packaging ~~influences~~ affects consumers' impulse buying ~~intentions. The findings show~~ intention. The research results indicate that both visual and verbal cues can significantly enhance impulse buying ~~intentions~~intention, with verbal cues having a stronger effect. ~~Visual cues enhance positive emotions and social presence, whereas verbal cues evoke stronger empathetic responses. The study validates the Stimulus-Organism-Response model , social presence theory, and empathy~~ This study validated the stimulus biological response model and emotion theory in the context of anthropomorphic design, enriching ~~related theoretical frameworks. Loneliness significantly moderates the relationship between anthropomorphic design and impulse buyingintentions. Higher levels of loneliness amplify the effects of anthropomorphic design. This suggests that businesses should tailor packagingand marketing strategies to address the psychological needs of lonely~~
