# Supplementary material for: Can anthropomorphic design in beverage packaging enhance impulse buying intention? Amazing visual and verbal cues!
Source: PLoS One. 2025 Jun 16;20(6):e0326186. doi: 10.1371/journal.pone.0326186 (PMC12169521; doi:10.1371/journal.pone.0326186)
Supplement: Attachment — Submitted filename: Response to Reviewers-5.12.docx [file pone.0326186.s003.docx]

Dear Editor and Reviewers,

We sincerely appreciate the time and effort you have dedicated to reviewing our manuscript. We are grateful for your constructive feedback, which has significantly improved the quality of our paper. Below, we provide our point-by-point responses to the reviewers’ comments and outline the revisions made to address their concerns. We have carefully addressed all the reviewers’ comments and believe the revised manuscript has been significantly improved. Thank you once again for your time and constructive feedback. We hope the current version meets the journal’s standards for publication. Should you have any further questions or require additional revisions, we would be happy to accommodate them.

Sincerely,

Yuyang Tian
College of Creative Arts, University Technology Mara (Uitm), Malaysia

**Response to comments：**

Reviewer #1:

I have no further comments on this version, and I wish you all the best with your publication and ongoing research.

We deeply appreciate your positive feedback and encouragement. Thank you for recognizing our efforts in revising the manuscript. We are delighted that you find the current version satisfactory and have no further comments.

Reviewer #2:

The authors have made great efforts to revise the paper as such the quality of the paper has been dramatically improved. I just have one concern: While the authors stated that they examined the CMV using ULMC method, I did not find the results in the manuscript. While the authors claimed that the results can be found in Lines 647 – 781, I still could not find them. Please follow the following approach to test CMV via ULMC and provide results in a table: Comparing the model fit indices such as CMIN/df, CFI, GFI, TLI, RMSEA, SRMR of the (1) measurement model, (2) measurement model with a ULMC, and (3) model with all items under 1 construct.

We sincerely thank you for your valuable suggestion regarding the Common Method Variance (CMV) analysis using the Unmeasured Latent Method Construct (ULMC) approach. We acknowledge that the presentation of the ULMC results in the original manuscript was not sufficiently clear. As per your recommendation, we have conducted a comprehensive ULMC analysis to assess CMV by comparing three models: the original measurement model, the measurement model with an added ULMC factor, and a single-factor model where all items load onto one construct. We have included a new table (Table 9) presenting the model fit indices (CMIN/df, CFI, GFI, TLI, RMSEA, SRMR) for all three models and added a clear interpretation of the results in the manuscript (now located in Section 4.6).
